# Dealing with Early-Onset Schizophrenia and the Role of Lurasidone: An Expert Opinion

**DOI:** 10.2174/011570159X385722250701114931

**Published:** 2025-07-15

**Authors:** Sergio De Filippis, Gianluca Serafini, Stefano Vicari, Antonio Vita, Benedetto Vitiello, Gabriele Masi

**Affiliations:** 1 Neuropsychiatric Clinic Villa von Siebenthal, Genzano di Roma, Italy;; 2 Department of Neuroscience, Rehabilitation, Ophthalmology, Genetics, Maternal and Child Health (DINOGMI), Section of Psychiatry, University of Genoa, Genoa, Italy;; 3 IRCCS Ospedale Policlinico San Martino, Genoa, Italy;; 4 Catholic University and Bambino Gesù Children’s Hospital, IRCCS, Rome, Italy;; 5 Spedali Civili di Brescia, University of Brescia, Brescia, Italy;; 6 University of Turin, Turin, Italy;; 7 Johns Hopkins University School of Public Health, Baltimore, MD, USA;; 8 IRCCS Stella Maris Foundation, Pisa, Italy

**Keywords:** Early Onset Schizophrenia EOS, lurasidone, antipsychotic drugs, EOS management, adolescence psychosis, psychiatric conditions

## Abstract

Compared to schizophrenia in adults, Early Onset Schizophrenia (EOS) features diagnostic, clinical, and therapeutic peculiarities that are the subject of ongoing discussion among psychiatrists and neuropsychiatrists. This article presents the outcomes of a meeting and a series of virtual roundtable discussions among specialists who validated practical recommendations for the diagnosis and management of EOS in light of recent literature. The identification of risk factors and prodromal symptoms, as well as the differentiation of EOS from other psychiatric conditions, is crucial for early detection. Timely identification enables the implementation of appropriate psycho-behavioural and pharmacological interventions and supports close monitoring of the developmental trajectories associated with EOS. The collaboration between the different professionals who deal with EOS patients and a therapeutic approach that allows a normal cognitive, sexual, and psychophysical development makes it possible to ensure the therapeutic alliance necessary for the optimal management of the disease over time. In a scenario that is complicated by negative prognostic factors, such as the late recognition of the disease, comorbid and latent psychiatric conditions, the increasingly widespread use of substances among adolescents, and a poor therapeutic adherence often due to antipsychotics side effects, a growing body of literature emphasizes the advantages of lurasidone in the treatment of EOS. Compared to other pharmacological agents commonly used in schizophrenia, lurasidone has been shown to intervene comprehensively and effectively against the positive and negative symptoms of EOS, with manageable side effects and the preservation of a good QoL.

## 1 INTRODUCTION

Early-onset Schizophrenia (EOS) presents between the ages of 13 and 18 years. It is estimated that about 12% of all cases of schizophrenia have their onset before the age of 18, while about 3% show their first signs and symptoms under 14 years of age [1]. Even though approximately 5% of children and adolescents display psychotic symptoms, only a few of them develop full-blown schizophrenia, which shows a prevalence of about 1% in the general population, with remarkable cross-country variations [2]. Delays in the identification of schizophrenia result in a longer Duration of Untreated Psychosis (DUP), which, along with a lack of treatment adherence, significantly contribute to the occurrence of Treatment-Resistant Schizophrenia (TRS) [3]. EOS entails diagnostic challenges as it occurs at a time when adolescents may also show autistic or attention deficit/hyperactivity disorder (ADHD) features that overlap with symptoms of impending schizophrenia [4].

Furthermore, substance use in vulnerable adolescents with psychotic symptoms may either confound clinicians or herald the progression to a schizophrenia spectrum disorder [5]. It is generally believed that EOS is more difficult to treat than the adult-onset form of the disease, given that currently available treatments are at best suboptimal and do not properly address the unmet needs of EOS [6]. Although multiple practice and treatment guidelines for EOS have been published, there is an urge for expert clinicians to meet and exchange their own ideas and expertise on how to implement research data and practice guidelines for EOS [7]. This publication aims to present the outcomes of a project in which Italian psychiatrists and neuropsychiatrists actively discussed current issues and unmet needs related to patients with EOS, as well as the role of lurasidone as a new therapeutic agent for the management of this condition.

## 2 METHODS

In May 2024, an Expert Panel of child/adolescent and adult psychiatrists was held to discuss diagnostic, clinical, and therapeutic aspects and unmet needs of EOS in the light of the latest scientific publications. This meeting resulted in 17 statements that the two project coordinators subsequently presented during three virtual round tables, held between July and September 2024, which involved about 30 child/adolescent and adult psychiatrists engaged in the frontline management of EOS patients in all Italian regions. These round tables provided the validation of the statements and their further implementation, based on the valuable clinical practice of all the participants.

## 3 DIAGNOSTIC AND CLINICAL ASPECTS

- EOS is a neurodevelopmental rather than a neurodegenerative disorder, the roots of which may be traced back to the pre-natal period (*e.g*., diet, infections, Maternal Immune Activation (MIA), toxic agents), often associated with impaired motor, language, cognitive, and social development, and with co-occurring neurodevelopmental disorders (*e.g*., autism spectrum disorder, ADHD, intellectual disability, learning disabilities).
- EOS presents significant challenges in differential diagnosis with respect to: Bipolar Disorder (BD) described by manic or mixed symptoms associated with psychotic symptoms misinterpreted as schizophrenic disorganization; Depressive Disorder (DD) featuring psychotic depressive symptoms, misinterpreted as negative symptoms, in the absence of cognitive impairment; Post-Traumatic Stress Disorder (PTSD) characterized by acute onset of flashbacks, derealisation/depersonalisation, suspiciousness, cognitive impairment, impulsiveness and alertness; and Obsessive-Compulsive Disorder (OCD) with schizotypal traits, such as lack of insight and bizarre obsessions.
- Psychiatric comorbidities should be actively and promptly identified to better tailor and define treatment.
- An extended DUP may be caused by diagnostic errors and delays, poor timing in treatment initiation, or lack of compliance.

Prenatal events can influence the development of psychotic disorders, in line with the “double hit” model. This model describes a “first hit” involving genetic and early environmental factors, such as maternal diet, infections, or exposure to toxic agents during gestation, which establish a baseline vulnerability. This may later be activated by a “second hit” during puberty or adolescence, triggered by factors, such as substance use, traumatic experiences, infectious diseases, and social isolation [8]. This framework also encompasses the concept of “pan-dysmaturation,” which refers to early premorbid alterations that result in motor, cognitive, and social deficits, often preceding the clinical onset of schizophrenia [9].

The differential diagnosis of EOS significantly overlaps with other major psychiatric disorders. For example, pediatric bipolar disorder, particularly in cases with mixed features and psychotic symptoms, is often misdiagnosed as schizophrenia; only over time and through follow-up does the correct diagnosis of BD become evident [10]. Similarly, patients with Major Depressive Disorder (MDD) may exhibit psychotic symptoms limited to the affective episode, without accompanying cognitive impairment. Their depressive mood can be misinterpreted as negative symptoms of schizophrenia [11]. Individuals with PTSD may present with repetitive and intrusive thoughts, flashbacks, derealization and depersonalization, cognitive alterations, hyperarousal, impulsivity, aggression, fears, and sleep disturbances, all of which can resemble psychotic features [12]. In OCD, patients may report bizarre or delusional-like obsessions, display poor insight, exhibit schizotypal traits, and may have a history or co-diagnosis of ASD or Tourette's syndrome [13].

Schizophrenia Spectrum Disorders (SSD), Autism Spectrum Disorder (ASD), Obsessive-Compulsive Disorder (OCD), and Bipolar Disorder (BD) exhibit significant genetic overlap, which partly explains the associations and comorbidities observed among these conditions. These shared genetic factors contribute to mixed clinical features and overlapping developmental pathways, particularly between ASD and SSD [14]. Schizotypal personality disorder may sometimes represent the evolution of ASD and, in some cases, it may progress to schizophrenia [15]. According to epidemiological findings, the diagnosis of OCD markedly increases the risk of both SSD (*i.e*., 12-fold) and BD (*i.e*., 13-fold) [16]. Half of the patients with EOS present with OCD symptoms, and these subjects have an earlier onset with a longer duration of psychotic symptoms [17]. OCD symptoms (*e.g*., contamination, symmetry control, cleaning, hoarding) and typical OCD comorbidities (*e.g*., hypochondria, tics, anorexia-bulimia) often precede and characterize the prodromal phase of EOS [18]. Conversely, few individuals with BD-I have psychotic disorders, making it necessary to implement broader diagnostic algorithms that associate psychosis with affective symptoms and cognitive aspects [19]. Therefore, neurodevelopmental disorders should be considered in their possible future trajectories as risk factors for psychosis itself [19].

The prodromal phase is characterized by behavioural changes, with the appearance of positive symptoms (*e.g*., ideas of reference, magical thinking, suspiciousness) [20]; cognitive symptoms (*e.g*., disturbances in attention, concentration, and planning) [20]; affective symptoms (*e.g*., unmotivated anxiety, depressive dysphoria, and irritability) and behavioural symptoms (*e.g*., impulsivity, aggression, and substance use) [20]. Furthermore, qualitative rather than quantitative changes in communication and affectivity, as well as a deterioration in global functioning, may be observed. However, clinical presentations are often heterogeneous and non-specific [20].

Scientific evidence shows that in EOS, and also in Very Early Onset Schizophrenia (VEOS), which presents before 1 year of age, a longer DUP correlates with a worse prognosis, and it is, therefore, crucial to carefully consider whether and how long to wait before pharmacological intervention [21].

## 4 PROGNOSTIC ASPECTS AND DISEASE TRAJECTORIES

- The Ultra High Risk (UHR) or At Risk of Mental States (ARMS) criteria used in adults should be carefully adapted to adolescents, who show more complex and unpredictable trajectories. These parameters may allow early detection of individuals at risk of developing EOS or with specific needs (*e.g*., psychosocial interventions). However, the low rate of conversion to psychosis does not justify the use of antipsychotics.
- Adolescents with schizophrenia frequently present with associated depressive or manic symptoms, along a dimensional clinical *continuum* between mood and psychotic disorders. A specific and timely assessment of these clinical conditions is also important, as it can influence treatment strategy and contribute to decreasing the risk of suicidal behaviours.
- In vulnerable individuals, drug use, including *cannabis*, cocaine, (meth)amphetamines, and hallucinogens, is associated with an increased risk of EOS occurrence at an earlier age, high recurrence, lower adherence to treatment, and worse prognosis.
- Positive prognostic factors of psychosis are a normal pre-morbid state, no family history of psychosis, an acute onset, the prevalence of positive symptoms, the occurrence of acute precipitating events (*e.g*., traumatic life events), an early and timely treatment, no drug use, and the presence of mood disorders in the person or within his/her family.

To characterize the red flags of the prodromal phase of psychosis, UHR criteria were identified. These include the Attenuated Psychotic Syndrome (APS), the Brief and Limited Intermittent Psychotic Symptoms (BLIPS) in the previous 12 months, and the description of “trait vulnerability” indicating genetic predisposition due to family history (*i.e*., Genetic Risk and Deterioration, GRD), or the presence of schizotypal personality disorder in the patient, or at least 30% functioning decline during the previous twelve months [22]. However, the criteria used in adults to delineate an ARMS *status* are not totally predictive in adolescents, who display more complex developmental trajectories [23]. The ARMS approach may also help recognize patients with “special needs”, in terms of preventative interventions, including monitoring of possible substance abuse, psycho-education, and social-cognitive stimulation [23].

The *continuum* between mood disorders and SSD has important clinical implications, particularly regarding the monitoring of suicide risk, which can reach 5% or higher when affective symptoms are present. Additionally, prognostic outcomes tend to be more favorable when bipolar or depressive disorders co-occur [24].

Substance Use Disorder (SUD) is common among young subjects with schizophrenia, and leads to a worse pre-morbid adaptation, an earlier schizophrenic onset, and more severe psychotic symptoms [25]. The comorbidity of schizophrenia and SUD reduces adherence to treatment, worsens outcomes, and increases the likelihood of relapse, hospitalization, and suicide attempts [25].

The perception of risk associated with Novel Psychoactive Substances (NPS) remains low, while the availability of new compounds continues to increase. Additionally, the age at first exposure to these substances has significantly decreased [25]. Scientific evidence indicates that the use of cannabis in adolescence is linked to a higher risk of developing EOS, particularly with early initiation, higher doses, prolonged use, and underlying psychiatric vulnerability [26]. Adults with psychosis who use cannabis during adolescence tend to have a poorer prognosis, characterized by more frequent relapses, increased hospitalizations, and lower treatment adherence [25].

## 5 EOS THERAPY: BASIC PRINCIPLES

- In the acute phase, the objective is the management of target symptoms (*e.g*., positive symptoms, aggressiveness), also by means of injectable formulations. However, a rapid switch to oral medication is desirable. The management of the post-acute phase depends on the efficacy of the treatment, its tolerability, changes in clinical presentation (*e.g*., mitigation of behavioural symptoms), and the possible onset or greater evidence of affective symptoms.
- Initial therapy is determined on the basis of the prevailing symptom dimensions, *i.e*., positive, negative, behavioural, cognitive, affective, as well as their degree of intensity. A secondary criterion is tolerability, based on specific features, such as baseline obesity or metabolic disorders.
- The dosage of the antipsychotic agent should be based on the minimum effective dose, with lower doses used to target negative symptoms and higher doses for positive or behavioral symptoms. Adverse effects typically depend on the specific agent, its dosage, the rate of titration, and potential drug interactions when multiple medications are prescribed. Dose-dependent side effects are sedation, cardiological signs (*e.g*., QTc prolongation), seizures, and hyperprolactinaemia, while extrapyramidal symptoms may often result from a rapid titration. Other side effects, such as agranulocytosis and an increase in appetite, are little affected by dosage and mostly depend on the mechanism of action of the chosen agent and the patient’s response.
- Therapeutic adherence depends on several critical factors, including intrinsic patient characteristics (*e.g*., history of drug abuse), disease features (*e.g*., presence of cognitive deficits), and the efficacy and tolerability of the chosen treatment. Additionally, adherence is influenced by the overall management of the patient with EOS, which involves establishing a strong patient-physician relationship and ensuring a smooth transition to adult healthcare services to maintain therapeutic continuity.
- Switching antipsychotic medications is recommended in cases of inadequate response, poor tolerability or compliance, or a high risk of long-term complications associated with the current agent. Cross-titration should be employed, involving a gradual tapering of the first drug alongside a stepwise increase of the second, thereby avoiding abrupt discontinuation and long-term polypharmacy. The specific switching strategies may vary depending on the drug class, such as ‘pines,’ ‘dones,’ and partial agonists.

The evidence supporting the effectiveness of psychological-behavioural intervention in individuals with EOS is rather low [27]. The use of antipsychotics is steadily increasing in all age groups, and these agents are increasingly prescribed off-label [28]. In the acute phase, the goal is the management of target symptoms, and this may require rapid titration or even the use of intramuscular-injection therapy, with a switch to oral therapy as soon as adherence has been successfully obtained [29]. Post-acute phase management depends on the efficacy of treatment over 6-8 weeks as well as tolerability. The adverse effects of antipsychotics vary according to the specific agent prescribed and may include weight gain and metabolic syndrome [30]; sedation, particularly during the initial phase or with rapid titration; extrapyramidal symptoms (EPS) and akathisia, especially at high doses or in sensitive individuals [30]; endocrine effects, such as hyperprolactinemia, which can cause amenorrhea, galactorrhea, libido changes, gynecomastia, and ejaculatory or erectile dysfunction [30]; cardiological effects including QTc prolongation; and hepatic effects, such as enzyme abnormalities and steatosis [30].

The most appropriate agent is chosen on the basis of the prevailing symptom dimension, and the lowest effective dose should be taken into account. In general, lower doses are administered when negative symptoms predominate, while higher amounts are given when positive symptoms and agitation are present [31]. Some antidepressant agents (*e.g*., fluoxetine, fluvoxamine, paroxetine) are often associated with antipsychotics, which may influence their metabolism and pharmacokinetics due to their inhibitory effects on cytochrome P450 isoenzymes [32].

Adherence to treatment depends on intrinsic patient factors (*e.g*., poor access to services, substance use, stigma, weak therapeutic alliance), illness characteristics (*e.g*., cognitive deficits, depression, lack of insight), and treatment-related factors (*e.g*., poor efficacy or tolerability) [33]. For acute patients, it is essential to optimize the timing of hospitalization and discharge while ensuring continuity of care. This continuity is crucial for preventing relapse and rehospitalization, controlling residual symptoms, and improving social functioning [31].

Switching drugs is recommended if the patient does not respond, in case of poor tolerability and compliance with the ongoing therapy, or if the risk of long-term complications with the current agent is too high [34]. As a general rule, cross-titration with a gradual tapering-off of the first drug while increasing the second without engaging in any unnecessary multi-drug therapy is preferred [34].

## 6 LURASIDONE IN EOS MANAGEMENT

- Lurasidone is used in the treatment of the first psychotic episode, both as a first-choice agent or when switching from other drugs due to lack of response, poor tolerability, or non-adherence; it is also prescribed in maintenance.
- Although the dosage of lurasidone in adolescents is 74 mg/day, treatment of severely ill individuals may require an increase to a maximum of 74 + 74 mg daily. Underdosing is likely to be interpreted as a lack of efficacy and can lead to untimely and unnecessary change of therapy.
  - Compared to other antipsychotics, lurasidone shows a highly favourable tolerability profile, and it is associated with minimal weight gain, negligible changes in blood glucose and lipid levels, no significant QTc interval prolongation, and less disruption of sexual and social life. These benefits contribute to good adherence and enhance the patient’s Quality of Life (QoL).
- In adolescents with psychosis and affective symptoms, lurasidone has proven to be effective in bipolar depression with a favourable cognitive and safety profile.

Lurasidone is an atypical antipsychotic that acts as a high-affinity antagonist at D2, 5-HT2A, and 5-HT7 receptors, a moderate-affinity agonist at 5-HT1A receptors, and shows negligible affinity for H1 and M1 receptors [35]. Its D2 antagonism underlies its efficacy in treating schizophrenia, while 5-HT2A antagonism helps improve negative symptoms and reduces Extrapyramidal Symptoms (EPS) and prolactin elevation. Additionally, lurasidone’s potent 5-HT7 receptor antagonism is linked to cognitive enhancement [36]. Clinically, lurasidone is primarily used for first-episode schizophrenia, maintenance therapy, and in patients with metabolic concerns or non-adherence to other medications. It should be taken with meals, preferably in the evening, to minimize nausea and maximize its tranquilizing effect [37]. Compared with placebo, lurasidone has shown efficacy in reducing the hostility/excitement dimension in acute schizophrenia, with an early symptom improvement, along with decreased negative symptoms and depressive/anxious type aspects, and a 148 mg/day dosage proved to be the most effective [38]. Similarly, all doses of lurasidone, particularly the higher ones (*i.e*., 120/160 mg/day), have demonstrated greater efficacy in reducing all scores of the Positive and Negative Syndrome Scale (PANSS) and the Clinical Global Impression Scale (CGI-S), with minimal metabolic changes [39].

Additionally, lurasidone, at doses of 148 mg and 74 mg, was significantly shown to reduce depressive symptoms and showed a decreased risk of treatment discontinuation [40]. A meta-review based on controlled studies including first and second-generation antipsychotics in children and adolescents, showed that lurasidone presents the best safety profile [41]. Compared to other agents, such as olanzapine, clozapine, risperidone, iloperidone, paliperidone, ziprasidone, and quetiapine, lurasidone results in less weight gain and milder changes in blood glucose and fats, as well as a lower incidence of seizures, QTc prolongation, and orthostatic hypotension [42]. Lurasidone ensures good treatment adherence as it indirectly improves QoL outcomes, including less impairment of sexual life and better social domain improvements compared to other therapeutic agents [43]. Two-year extension studies confirmed that lurasidone leads to continuous improvement in functioning and QoL in acutely ill adolescent and young adult responders and remitters, with slight changes in metabolic parameters and weight gain, consistent with expected changes in individuals whose developmental trajectories are still evolving [44].

Furthermore, lurasidone enabled the normalization of prolactin levels when switching from risperidone, proving to be a valuable therapeutic agent, especially in young individuals with dysregulated metabolism [45]. Regarding adverse drug reactions (ADRs), a retrospective, observational, multicenter study by Fagiolini *et al*. evaluated treatment persistence with lurasidone in adult psychotic patients at 6 and 18 months. ADRs were reported in 14.63% of the population, none of which were classified as serious. The most commonly reported ADRs included akathisia (7.32%), extrapyramidal symptoms (EPS) (4.88%), hyperprolactinemia (2.44%), restlessness (2.44%), and galactorrhea (2.44%). These findings are consistent with more recent studies, which have identified similar ADRs, including sleep disturbances, occurring at comparable incidence rates during lurasidone treatment [46-57].

## CONCLUSION

Compared to adults with schizophrenia, patients with EOS exhibit distinct clinical features that can delay diagnosis and treatment. However, early intervention has been shown to be beneficial, helping to slow clinical deterioration and prevent relapse, as supported by recent evidence [58-67]. Therefore, comprehensive management of young individuals with psychosis, along with the use of medications that do not negatively impact their quality of life, is of paramount importance. Growing scientific evidence suggests that lurasidone aligns well with these treatment goals and may represent a valuable therapeutic option to enhance adherence and support long-term management in individuals with EOS.

## STUDY LIMITATIONS

The discussion among the experts highlighted the extreme geographical variability in the treatment of EOS, and the statements hereby presented need to be tested in the specific contexts in which specialists work. The management of patients with EOS, which has been rationalised in this publication, clashes with daily clinical practice, which is limited by various factors, including the organisational and human resources available for the diagnosis, treatment, and long-term management of overall psychiatric disorders.

## LIST OF ABBREVIATIONS

ADHD: Attention-Deficit/Hyperactivity Disorder
ADRs: Adverse Drug Reactions
APS: Attenuated Psychotic Syndrome
ARMS: At-Risk Mental State
BD: Bipolar Disorder
BLIPS: Brief Limited Intermittent Psychotic Symptoms
CGI-S: Clinical Global Impression Scale
DD: Depressive Disorder
DUP: Duration of Untreated Psychosis
EOS: Early-Onset Schizophrenia
EPS: Extrapyramidal Symptoms
GRD: Genetic Risk and Deterioration
MDD: Major Depressive Disorder
MIA: Maternal Immune Activation
NPS: Novel Psychoactive Substances
OCD: Obsessive-Compulsive Disorder
PANSS: Positive and Negative Syndrome Scale
PTSD: Post-Traumatic Stress Disorder
QoL: Quality of Life
SSD: Schizophrenia Spectrum Disorders
SUD: Substance Use Disorder
TRS: Treatment-Resistant Schizophrenia
UHR: Ultra-High Risk
VEOS: Very Early Onset Schizophrenia
